# Hydrogel Glucose Sensor with *In Vivo* Stable Fluorescence Intensity Relying on Antioxidant Enzymes for Continuous Glucose Monitoring

**DOI:** 10.1016/j.isci.2020.101243

**Published:** 2020-06-06

**Authors:** Jun Sawayama, Teru Okitsu, Akihiro Nakamata, Yoshihiro Kawahara, Shoji Takeuchi

**Affiliations:** 1 Institute of Industrial Science, The University of Tokyo, 4-6-1 Komaba, Meguro-ku, Tokyo, Japan; 2 Graduate School of Information Science and Technology, The University of Tokyo, 7-3-1 Hongo, Bunkyo-ku, Tokyo, Japan; 3 International Research Center for Neurointelligence (WPI-IRCN), The University of Tokyo Institutes for Advanced Study, The University of Tokyo, 7-3-1 Hongo, Bunkyo-ku, Tokyo, Japan

**Keywords:** Biochemical Mechanism, Glycobiology, Medical Device, Sensor

## Abstract

Hydrogel glucose sensors with boronic acid-based fluorescence intensity theoretically hold promise to improve *in vivo* continuous glucose monitoring (CGM) by facilitating long-lasting accuracy. However, these sensors generally degrade after implantation and the fluorescence intensity decreases immediately over time. Herein, we describe a hydrogel glucose sensor with *in vivo* stability based on boronic acid-based fluorescence intensity, integrating two antioxidant enzymes, superoxide dismutase (SOD), and catalase. These protected the arylboronic acid from being degraded by hydrogen peroxide *in vitro* and preserved the boronic acid-based fluorescence intensity of the hydrogel glucose sensors in rats for 28 days. These antioxidant enzymes also allowed the hydrogel glucose sensor attached to a homemade semi-implantable CGM device to trace blood glucose concentrations in rats for 5 h with the accuracy required for clinical settings. Hydrogel glucose sensors with boronic acid-based fluorescence intensity containing SOD and catalase could comprise a new strategy for *in vivo* CGM.

## Highlights

• The arylboronic acids of hydrogel glucose sensors are sensitive to cleavage by ROS
• The antioxidant enzymes suppress the degradation of fluorescence effectively *in vivo*
• The developed sensor performs CGM with the accuracy required for clinical settings

## Graphical Abstract

**Figure:**
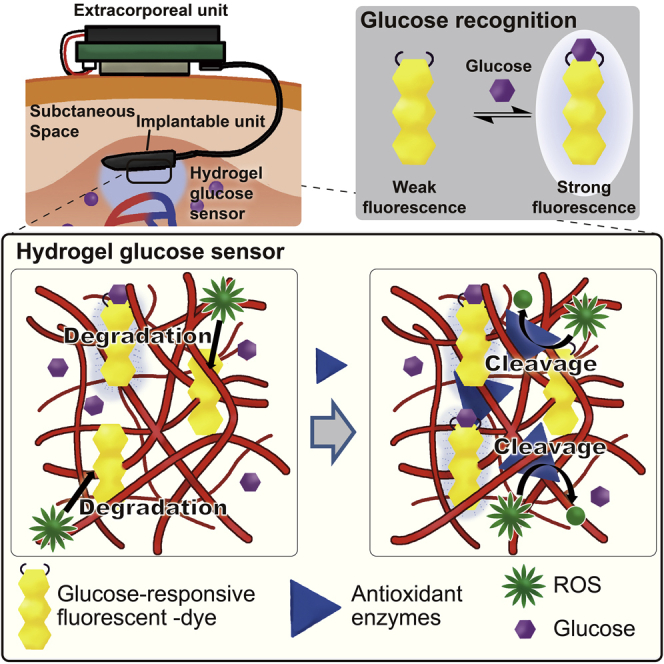

## Introduction

Diabetes mellitus is defined as a group of metabolic disorders characterized by high blood glucose concentrations (Cho et al., 2018). To control these levels though insulin therapy for the treatment of diabetes mellitus, it is important to accurately and continuously monitor blood glucose concentrations, because they are used to determine the dose of insulin for injection and the glucose levels could change after insulin injection, from levels representative of hyperglycemia to those associated with hypoglycemia, in a short period of time (Nathan et al., 1993). For continuous glucose monitoring (CGM) in clinical settings, enzyme-based glucose sensors are currently the standard, but they lack accuracy in determining insulin doses (Kovatchev et al., 2015). This drawback is because the enzymes that react with glucose are inactivated during monitoring and the sensor's current value corresponding to a certain glucose concentration changes over time (Harris et al., 2013, Wilson and Turner, 1992). One approach to overcome such limitations of currently standard CGM is to replace the sensors with hydrogel glucose sensors based on boronic acid-based fluorescence intensity (Mortellaro and DeHennis, 2014, Colvin and Jiang, 2013, Hansen and Christensen, 2013, Shibata et al., 2010, Heo et al., 2011). Boronic acids maintain their activities during the detection of glucose, and the fluorescence intensity corresponding to a certain glucose concentration is not expected to change. Despite this expectation, immediately after the sensors are implanted *in vivo*, they degrade and fluorescence intensity decreases.

It is experimentally known that additives with antioxidant activity for boronic acid-based fluorescence glucose sensors suppress the degradation of *in vivo* fluorescence (Colvin and Jiang, 2013). However, the mechanism underlying the *in vivo* decrease in the fluorescence intensity of hydrogel glucose sensors has not been clarified yet. Understanding this mechanism is expected to lead to further improvements in the *in vivo* sustainability of hydrogel glucose sensors.

In this study, we clarified the cause of the *in vivo* degradation of hydrogel glucose sensors with boronic acid-based fluorescence intensity by observing changes in the chemical structures. Then, we developed a hydrogel glucose sensor containing a highly active antioxidant reagent. We subsequently evaluated the effects of the antioxidant additives in the hydrogel glucose sensor by implanting this device into rats. To demonstrate the efficacy of the hydrogel glucose sensors containing antioxidant additives, we performed CGM using the implantable fluorescent device and evaluated its accuracy for this application.

## Results and Discussion

### *In Vivo* Fluorescence Degradation of the Hydrogel Glucose Sensor and Degradation Mechanism

**Figure 1:**
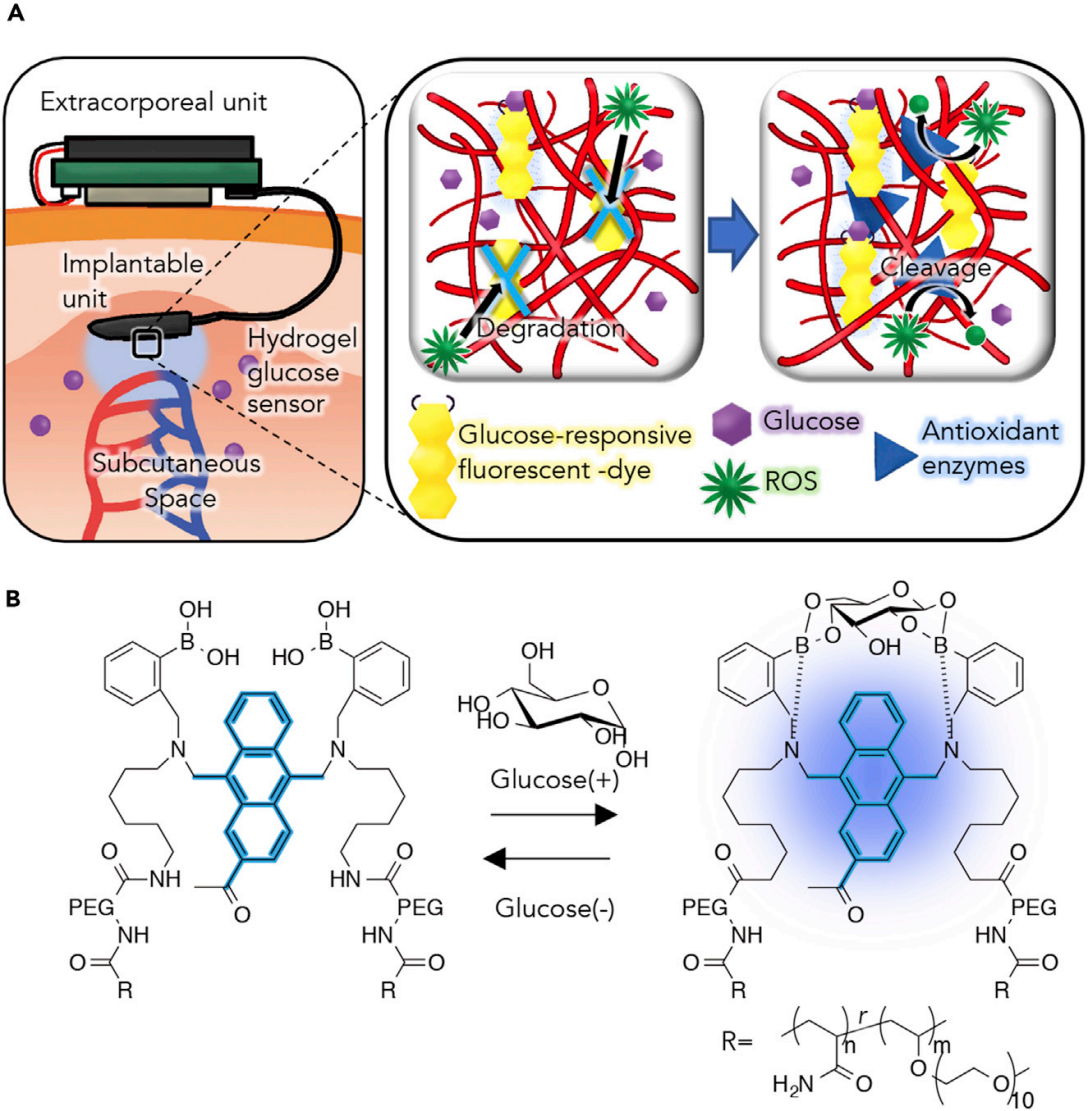
Schematic Illustration of the CGM and Mechanism of Glucose Recognition (A) Schematic illustration of the *in vivo* stable fluorescent-based hydrogel glucose sensor. (B) Glucose response mechanism of GF dye. In the absence of glucose molecules, the fluorescence of the anthracene is quenched. When glucose molecules bind to the arylboronic acid, the fluorescence intensity increased.

**Figure 2:**
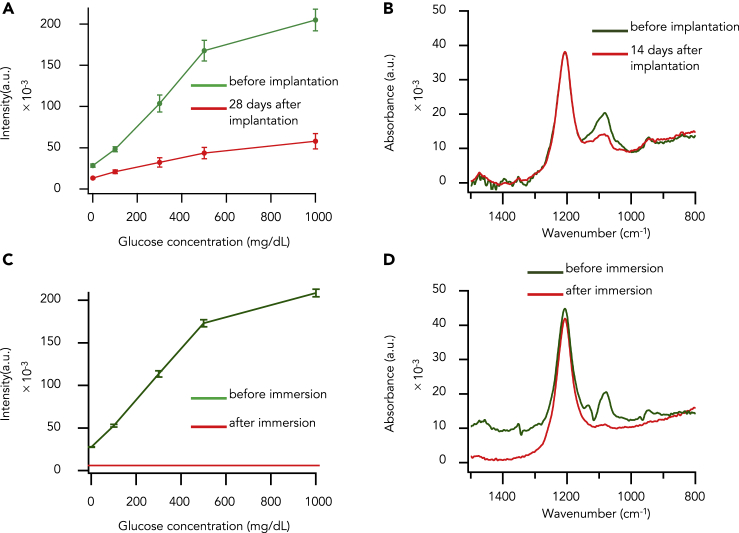
Fluorescence Degradation of the Hydrogel Glucose Sensor (A) Curves of relationship of fluorescent intensity and glucose concentration sensor hydrogels about before (green) and 28 days after implantation (red). Data are presented as the mean ± SD (n = 3). (B) IR spectra of sensor hydrogels before implantation (green) and 14 days after implantation (red). (C) Curves of relationship of fluorescent intensity and glucose concentration of sensor hydrogels about before (green) and immersion into the 35 μM H_2_O_2_ (red). Data are presented as the mean ± SD (n = 3). (D) IR spectra of sensor hydrogels before immersion (green) and after immersion (red). See also Figures S1 and S2.

For CGM, we previously developed a hydrogel glucose sensor with boronic acid-based fluorescence intensity (Figure 1A) (Shibata et al., 2010, Heo et al., 2011), in which the number of glucose molecules binding to two arylboronic acids determines the fluorescence intensity of anthracene through electron transfer from the adjacent nitrogen atoms; specifically, more glucose molecules are reflected by an increase in fluorescence intensity (Figures 1B). To confirm whether *in vivo* fluorescence degradation, which has been observed in conventional boronic acid-based fluorescent glucose sensors (Heo et al., 2011), would be reproduced in our glucose sensor, we performed the following steps. We fabricated our hydrogels of the fluorescent glucose sensor into a plate shape, implanted one plate-shaped hydrogel into each subcutaneous space of three rats, retrieved the hydrogels 28 days after implantation, and compared the fluorescence intensities of the hydrogels before and 28 days after implantation. We found that the fluorescence intensities of the hydrogels were reduced significantly at all glucose concentrations after implantation when compared with those before implantation (Figures 2A and S1A); furthermore, the rates of reduction were as follows: 53.5 ± 0.5% at 0 mg dL^−1^ of glucose, 56.2 ± 7.1% at 100 mg dL^−1^, 68.3 ± 9.1% at 300 mg dL^−1^, 73.6 ± 6.3% at 500 mg dL^−1^, and 71.4 ± 6.6% at 1,000 mg dL^−1^.

Next, to elucidate whether this *in vivo* fluorescence degradation was ascribed to structural disorders of our hydrogel glucose sensors, we analyzed their chemical structures before and 14 days after implantation using Fourier transform infrared (FTIR) spectroscopy. We found that the band at 1,080 cm^−1^ that was assigned to boron-carbon stretching bonds in the arylboronic acids (Faniran and Shurvell, 1968) was lower after implantation than before implantation (Figure 2B). These results clearly showed that the boronic acids of our hydrogel glucose sensors are cleaved *in vivo* and indicated that this cleavage causes the degradation of *in vivo* fluorescence.

We were then interested in what caused the cleavage of arylboronic acids *in vivo* and presumed that reactive oxygen species (ROS) might be responsible because it was reported that they exist *in vivo* (Lefer and Granger, 2000) and that they oxidize and cleave boronic acid (Dickinson and Chang, 2011, Kalyanaraman et al., 2012). To address this through *in vitro* experiments, we adopted hydrogen peroxide as the only useful ROS for these experiments, as this compound exists ubiquitously *in vivo* (Kalyanaraman et al., 2012, Kalyanaraman et al., 2012) and would be sufficiently stable *in vitro* for the duration of the experiments (Gomes et al., 2005, Miller et al., 2007, Chang et al., 2004). We then kept our sensor hydrogels immersed in a 35-μM hydrogen peroxide aqueous solution for 30 min and compared the fluorescence intensities and the chemical structures of the hydrogels before and after immersion. We found that the fluorescence intensities of the hydrogels diminished to almost zero at all glucose concentrations after immersion (Figures 2C and S1B) and that the FTIR band assigned to the boron-carbon stretching bonds of the arylboronic acids almost disappeared after immersion (Figure 2D). These results show that the arylboronic acids of our hydrogel glucose sensors are sensitive to cleavage by physiological concentrations of ROS (Figure S2A), indicating that such compounds existing *in vivo* might cause the *in vivo* fluorescence degradation of our hydrogel glucose sensors.

### Prevention of *In Vivo* Fluorescence Degradation of the Hydrogel Glucose Sensor Using Antioxidant Enzymes

To prevent this degradation by ROS *in vivo*, we modified our hydrogel glucose sensor to contain two antioxidant enzymes, superoxide dismutase (SOD) and catalase (Figure S2B). This strategy was based on the following two facts. First, these two antioxidant enzymes are thought to inactivate representative ROS *in vivo* including superoxide radical (O_2_^•^), hydrogen peroxide (H_2_O_2_), superoxide radical anion (O_2_^•−^), hypochlorous (HOCl), peroxynitrite (ONOO^−^), perhydroxyl radical (HO^•2^), and nitrogen dioxide (NO_2_). This is because SOD and catalase directly inactivate superoxide radicals and hydrogen peroxide, respectively, and these two antioxidant enzymes indirectly inactivate other ROS since superoxide radical and hydrogen peroxide serve as the precursors of superoxide radical anion (O_2_^•−^), hypochlorous (HOCl), peroxynitrite (ONOO^−^), perhydroxyl radical (HO^•2^), and nitrogen dioxide (NO_2_) (Winterbourn, 2008; Forman et al., 2010). Second, the inactivation of superoxide radical and hydrogen peroxide by SOD and catalase can be expected to precede the decomposition of the arylboronic acids by these two ROS in the hydrogel glucose sensor, because the two antioxidant enzymes inactivate these two ROS faster than these two ROS decompose arylboronic acids (Foyer et al., 1994). This assumption is based on the following information: the reaction rate of catalase and hydrogen peroxide is higher than that of hydrogen peroxide and arylboronic acid (2.0 × 10^7^ M^−1^s^−1^[Beers and Sizer, 1952] versus 2.5 M^−1^s^−1^[Sikora et al., 2009]). Moreover, the reaction rate of SOD and superoxide radical is higher than that of superoxide radical and arylboronic acid, because the former is 2.0 × 10^9^ M^−1^s^−1^(Kono and Fridovich, 1982, Forman and Fridovich, 1973) and the latter would be <0.5 × 10^−2^ M^−1^s^−1^, as deduced from previous studies showing that superoxide radical is >500-fold less responsive to arylboronic acid than hydrogen peroxide (Miller et al., 2007, Chang et al., 2004).

**Figure 3:**
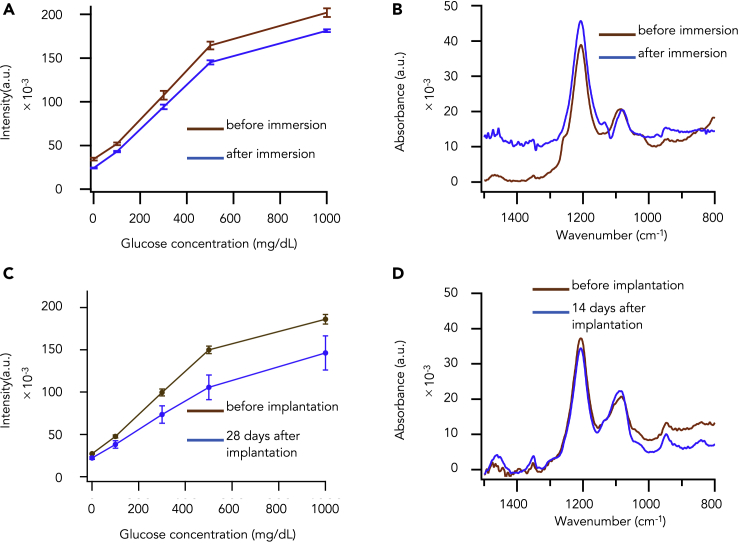
Prevention of Fluorescence Degradation of the Hydrogel Glucose Sensor Containing Antioxidant Enzymes (A) Curves of relationship of fluorescent intensity and glucose concentration of hydrogel sensor containing antioxidant enzymes about before (brown) and after immersion into the 35 μM H_2_O_2_ (blue). Data are presented as the mean ± SD (n = 3). (B) IR spectra of hydrogel before immersion (brown) and after immersion (blue). (C) Curves of relationship of fluorescent intensity and glucose concentration of hydrogel sensor containing antioxidant enzymes about before (brown) and 28 days after implantation (blue). Data are presented as the mean ± SD (n = 3). (D) IR spectra of hydrogel before implantation (brown) and 14 days after implantation (blue). See also Figure S3, Tables S1 and S2.

Through *in vitro* experiments, we evaluated the effect of these antioxidant enzymes on the fluorescence degradation of the hydrogel glucose sensors. The hydrogel glucose sensor containing antioxidant enzymes was immersed for 30 min in 200 μM hydrogen peroxide aqueous solution. We found that the hydrogels retained 88.2 ± 3.2% of original fluorescence intensities at various glucose concentrations after immersion (Figures 3A and S3A). Subsequently, we analyzed the chemical structures of hydrogel glucose sensors containing antioxidant enzymes before and after immersion by FTIR. We found that, after immersion, the band at 1,080 cm^−1^ was almost the same as that before (Figure 3B). These results suggest that the antioxidant enzymes protect the arylboronic acid from being degraded by hydrogen peroxide and allow the hydrogel glucose sensors to preserve fluorescence intensities *in vitro*.

Next, through rat experiments, we confirmed the *in vivo* contribution of the antioxidant enzymes to the preservation of fluorescence intensities in hydrogel glucose sensors. We fabricated plate-shaped hydrogels containing antioxidant enzymes, implanted them into the subcutaneous spaces of three rats, retrieved the hydrogels after 28 days, and compared the fluorescence intensities of the hydrogels with those before implantation. We found that the fluorescence degradation of the hydrogels was suppressed at various glucose concentrations (Figures 3C and S3B) and that the rates of reduction were as follows: 18.4 ± 2.5% at 0 mg dL^−1^ of glucose, 20.0 ± 6.2% at 100 mg dL^−1^, 26.3 ± 7.4% at 300 mg dL^−1^, 29.8 ± 7.7% at 500 mg dL^−1^, and 21.6 ± 9.1% at 1,000 mg dL^−1^. Furthermore, the bands at 1,080 cm^−1^ of FTIR were almost the same before and after implantation (Figure 3D). These results indicate that the antioxidant enzymes suppress the degradation of fluorescence effectively *in vivo* and *in vitro* by protecting arylboronic acid from being degraded by ROS such as hydrogen peroxide.

To investigate the biocompatibility of the hydrogel glucose sensor, we have asked a contract research organization to perform a histopathological examination. As the results, no remarkable change was observed at the tissue of liver, testis, epididymis, and sciatic nerve 28 days after implantation of the hydrogels into the back of Slc:SD rats (Table S1). Even in the subcutaneous spaces, we found that the biocompatibility of the hydrogel glucose sensor was equivalent to the one of polyurethane, which is generally used in implantable devices (Table S2).

### Continuous Glucose Monitoring in Rats Using the Hydrogel Glucose Sensor Containing Antioxidant Enzymes

To apply the hydrogel glucose sensor containing antioxidant enzymes to CGM *in vivo*, we first developed a semi-implantable device that can be equipped with the hydrogel glucose sensor (Figure S4A). The device was composed of two units; one was an implantable unit to monitor the fluorescence intensity of the hydrogel glucose sensor, whereas the other was an extracorporeal unit to wirelessly transfer the fluorescence intensities in real time to the PC for recording (Figure S4C). In the implantable unit, the hydrogel glucose sensor was located on polydimethylsiloxane (PDMS) covering an electronic circuit (Figure S5B) that was integrated with a LED light, as well as a photodiode (PD) (Figures S5A and S5C). The validity of this device was confirmed both by placing a fluorescence standard with a main emission peak of 460 nm at the location where the hydrogel glucose sensor was supposed to be placed and by subsequently confirming that the fluorescence intensity of the standard over 72 h was constantly approximately 1,000 and within 0.4% of the coefficient of variation (Figure S4B). Next, we developed an algorithm to convert the time-series data of the fluorescence intensities of the hydrogel glucose sensors recorded in the PC to time-series glucose concentrations. The algorithm mainly included a logarithmic function that was derived from an exponential approximation to plot a calibration curve, showing the relationship between the fluorescence intensity and glucose concentration. The sample data for the calibration curves were obtained by measuring individual fluorescence intensities of both hydrogel glucose sensors alone and those containing antioxidant enzymes *in vitro* by putting the implantable units into standard glucose solutions at concentrations of 0, 100, 300, 500, and 1,000 mg dL^−1^. Through these procedures, for either hydrogel glucose sensors alone or those containing antioxidant enzymes, we found that the fluorescence intensity increased as the glucose concentration was elevated and vice versa (Figures S6A and S6C). Moreover, the fluorescence intensities and glucose concentrations corresponded at a 1:1 ratio (Figures S6B and S6D). Based on these results, we determined that the semi-implantable device was successfully developed and ready for evaluation as a hydrogel glucose sensor to continuously monitor glucose concentrations *in vivo*.

**Figure 4:**
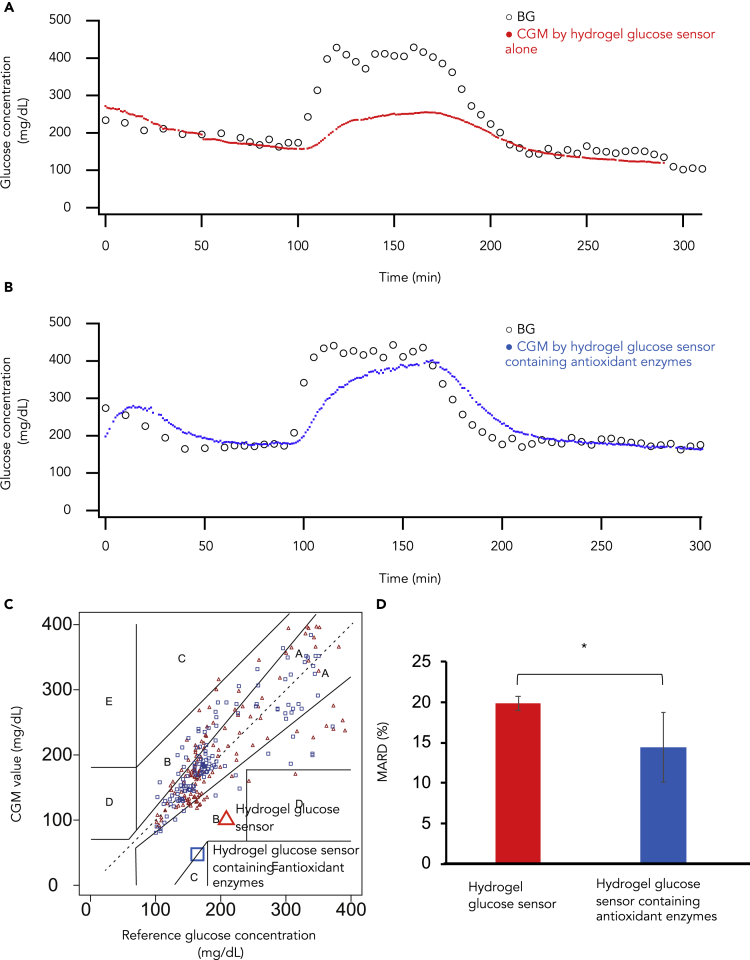
Hyperglycemia Clamp Test in Rats under Anesthesia (A) Continuous glucose monitoring used the implantable fluorescent device equipped with hydrogel glucose sensor alone. (B) Continuous glucose monitoring used the implantable fluorescent device equipped with hydrogel glucose sensor containing antioxidant enzymes. (C) The Clarke Error Grid Analysis of hydrogel glucose sensor (red) and hydrogel glucose sensor containing antioxidant enzymes (blue) (N = 3). (D) MARD of continuous glucose monitoring using hydrogel glucose sensors (red) and hydrogel glucose sensor containing antioxidant enzymes (N = 3). ∗p < 0.05. See also Figures S4–S6.

Subsequently, we used rats to test the *in vivo* functions of hydrogel glucose sensors and intended to evaluate the influence of antioxidant enzymes in the hydrogel glucose sensors on the accuracy of CGM. Specifically, we first placed the hydrogel glucose sensor accompanied by the implantable units into the subcutaneous spaces of rats under anesthesia. Immediately, we started to measure the fluorescence intensities of hydrogel glucose sensors using the semi-implantable device every minute, as well as blood glucose concentrations using a self-measurement of blood glucose device every 5 min. One hundred minutes after the start of the experiments, the rats underwent hyperglycemic clamp tests. We found that the hydrogel glucose sensor alone failed to trace blood glucose concentrations within the hyperglycemic range (Figure 4A) but the one containing antioxidant enzymes succeeded in tracing blood glucose concentrations in both euglycemic and hyperglycemic ranges (Figure 4B). Next, we performed Clarke error grid analysis by plotting the calculated glucose concentrations against the reference glucose concentrations of both the hydrogel glucose sensors alone and those containing antioxidant enzymes after performing three sets of *in vivo* experiments using rats for each sensor. We found that 74% of the data belonged to Zone A, 24% to Zone B, 1% to Zone C, and 1% to Zone D for the hydrogel glucose sensors alone and that 77% of the data belonged to Zone A, 22% to Zone B, 1% to Zone C, and 0% to Zone D for the hydrogel glucose sensors containing antioxidant enzymes (Figure 4C). Furthermore, we found that the MARD (mean absolute relative difference): Accuracy of CGM Systems (Kovatchev et al., 2015, Reiterer et al., 2017) for the hydrogel glucose sensor alone was significantly larger than that for the hydrogel glucose sensor containing antioxidant enzymes (19.9 ± 0.84% versus 14.5 ± 4.2%, p < 0.05; Figure 4D). These results show that antioxidant enzymes allow hydrogel glucose sensors to monitor the glucose concentrations accurately *in vivo*.

### Conclusion

We developed the first prototype of a hydrogel glucose sensor with boronic acid-based fluorescence intensity incorporating antioxidant enzymes based on our findings on the mechanism underlying the *in vivo* degradation of arylboronic acid. To our knowledge, here, we report for the first time the mechanism underlying the degradation of arylboronic acid; specifically, ROS, existing *in vivo*, are most likely the main factors that contribute to this. In addition to the development of the sensor prototype, we demonstrated that the developed sensor can perform CGM in rats with the accuracy required for clinical settings even over several hours; however, the conventional sensor without antioxidant enzymes could not achieve this. Although the new hydrogel glucose sensor with boronic acid-based fluorescence intensity offers considerable promise for diabetes treatment, the sensor requires further developments such as improvements to extend the period of validity and extensive validation using larger experimental animals. Ultimately, such CGM with long-lasting, highly accurate hydrogel glucose sensors presents new possibilities not only for diabetes management but also for diabetes therapy, especially by promoting the development of a next-generation artificial pancreas.

### Limitations of the Study

Some limitations to the findings of this study must be acknowledged. The size of implantable unit of our device was 8 × 20 × 6 mm^3^. In clinical use, the implantable unit should be downscaled at least less than 0.5 mm in diameter to reduce the invasiveness. To fabricate the miniaturized implantable unit, micromachining technology such as MEMS or microfluidic technology is considered to be effective. One additional limitation of the study is using polyacrylamide gel for immobilizing GF-dye. The polyacrylamide gel probably induces foreign body reaction after implantation and is eventually encapsulated by living cells and extracellular matrices. One approach to solve this problem can be to replace the polyacrylamide gel with another type of hydrogel that is more biocompatible having the hydrophilicity and low protein adsorption affinity such as polyethylene glycol.

### Resource Availability

#### Lead Contact

Further information and requests for resources and reagents should be directed to and will be fulfilled by the Lead Contact, ShojiTakeuchi (takeuchi@iis.u-tokyo.ac.jp).

#### Materials Availability

Our study did not generate any new unique reagents.

#### Data and Code Availability

Our study did not report any unpublished custom code, or software, or algorithm.

## Methods

All methods can be found in the accompanying Transparent Methods supplemental file.

## Supplemental Information

Document S1. Transparent Methods, Figures S1–S6, and Tables S1 and S2
