# Supplementary material for: Hydrogel Glucose Sensor with In Vivo Stable Fluorescence Intensity Relying on Antioxidant Enzymes for Continuous Glucose Monitoring
Source: iScience. 2020 Jun 6;23(6):101243. doi: 10.1016/j.isci.2020.101243 (PMC7306611; doi:10.1016/j.isci.2020.101243)
Supplement: Document S1. Transparent Methods, Figures S1–S6, and Tables S1 and S2 [file mmc1.pdf]

iScience, Volume *23*

Supplemental Information

Hydrogel Glucose Sensor with *In Vivo* Stable Fluorescence Intensity Relying on Antioxidant Enzymes for Continuous Glucose Monitoring

Jun Sawayama, Teru Okitsu, Akihiro Nakamata, Yoshihiro Kawahara, and Shoji Takeuchi

Supplemental Information

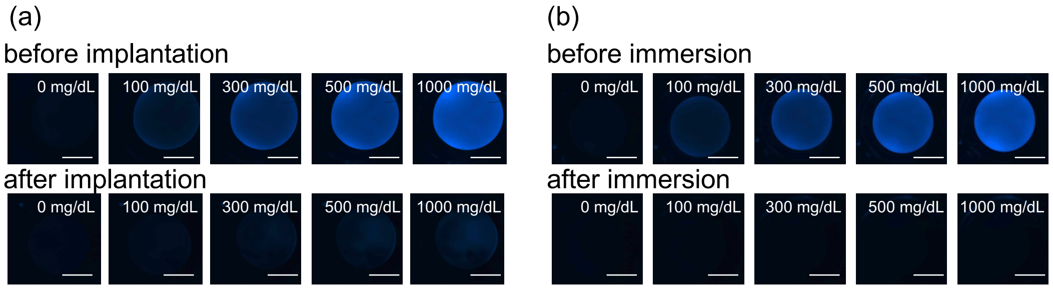

Figure S1 Related to Figure 2. Fluorescent image of hydrogel glucose sensors

(a) Fluorescent image of hydrogel sensors into 0, 100, 300, 500, 1000 mg/dL glucose solution, respectively. Upper column is before implantation and lower column is 28 days after implantation. (b) Upper column is before immersion and lower column is after immersion into the 35 μM H_2O_2.

(a)

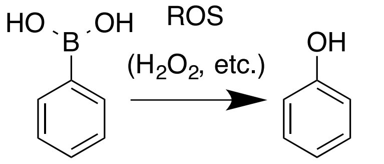

(b)

$$2\,H_2O_2 \xrightarrow{\text{Catalase}} O_2 + 2\,H_2O$$

$$2\,O_2^- + 2\,H^+ \xrightarrow{\text{SOD}} O_2 + 2\,H_2O_2$$

Figure S2 Related to Figure 3. Mechanism of the *in vivo* degradation of GF-dye and cleavage of ROS by antioxidant enzymes

(a) Oxidation scheme of arylboronic acid that exposed to reactive oxygen species (ROS)such as hydrogen peroxide. (b) Mechanism of cleavage of ROS by catalase and superoxide dismutase (SOD) that mixed into GF-gel.

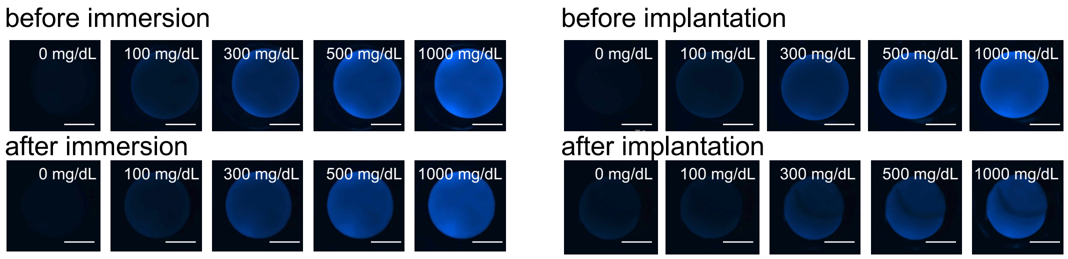

Figure S3 Related to Figure 3. Fluorescent image of hydrogel glucose sensors containing antioxidant enzymes

(a) Fluorescent image of hydrogel sensor containing antioxidant enzymes into 0, 100, 300, 500, 1000 mg/dL glucose solution, respectively. Upper column is before immersion,and lower column is after immersion into the 35 μM H_2O_2. (b) Upper column is before implantation,and lower column is 28 days after implantation.

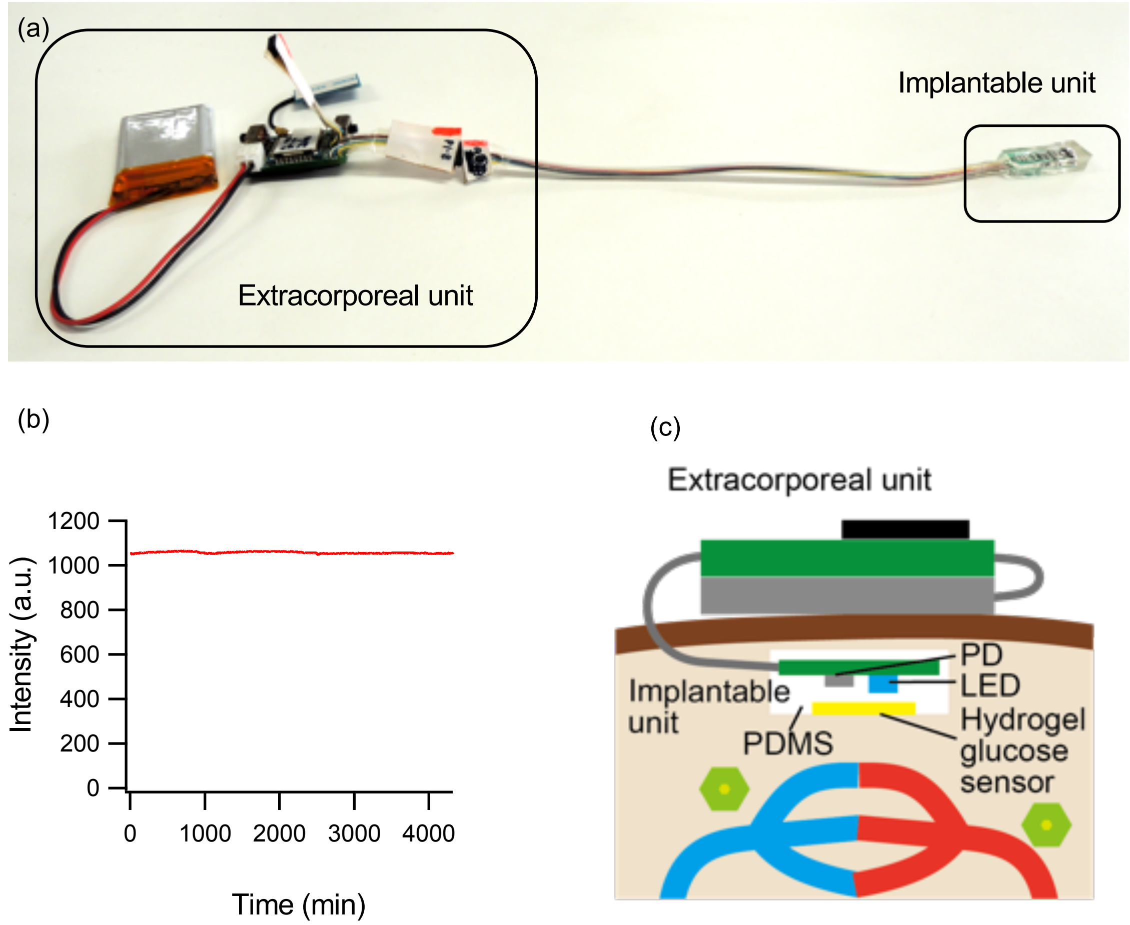

Figure S4 Related to Figure 4. Implantable fluorescence device

(a) total image, (b) stability of fluorescent device, and (c) schematic image of implantation.

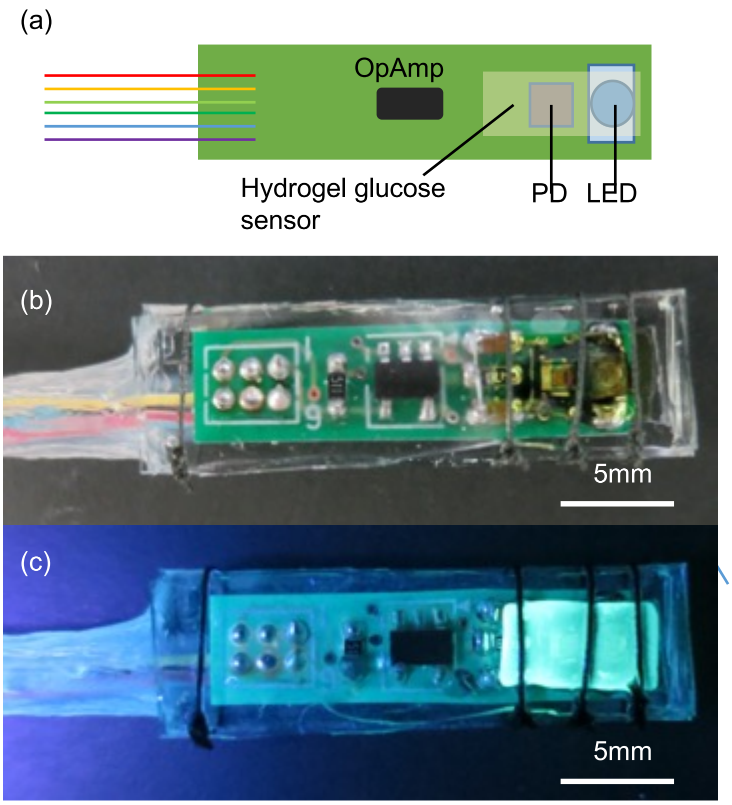

Figure S5 Related to Figure 4. Implantable unit equipped with hydrogel glucose sensor

(a) schematic image of implantable unit, (b) image under the room light, and (c) image under the irradiation of UV lamp.

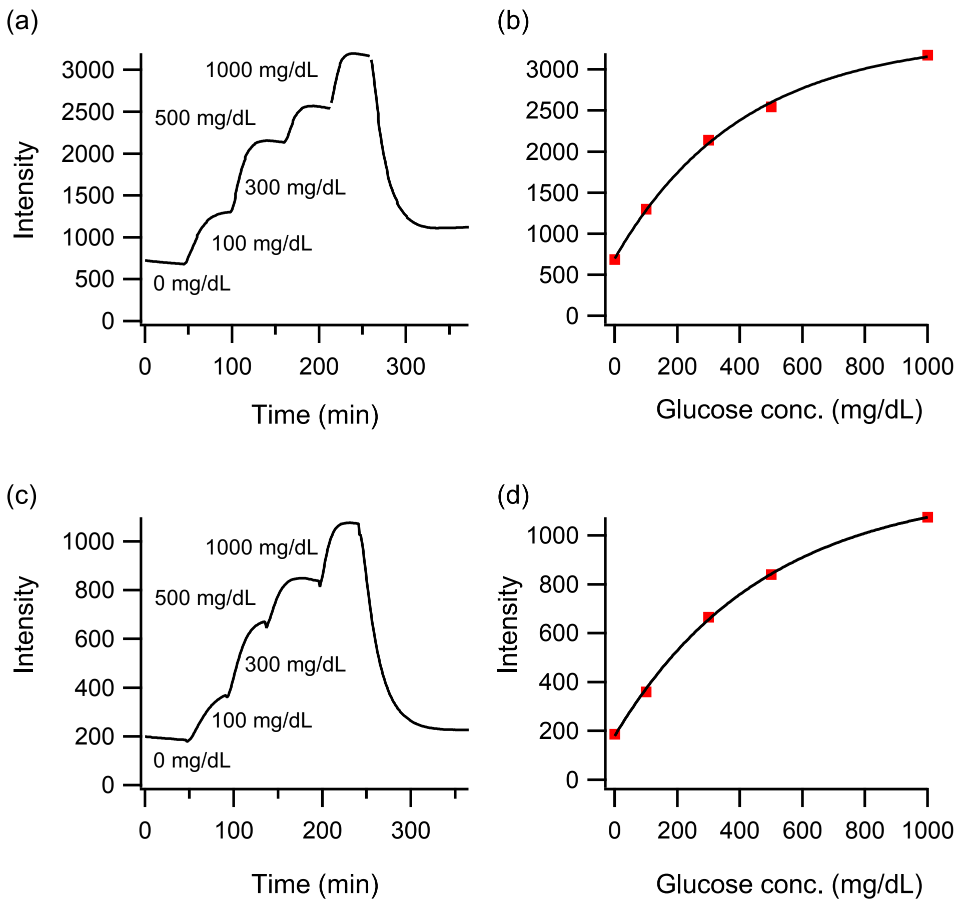

Figure S6 Related to Figure 4. Typical glucose-monitoring trace obtained *in vitro* experiment. and relationship between fluorescence intensity and glucose concentration

(a,b) hydrogel glucose sensor , and (c,d) hydrogel glucose sensor containing antioxidant enzymes.

Tissue	Observation	Animal No.	entry 1	entry 2	entry 3	entry 4	entry 5
Liver			-	-	-	-	-
Testis							
	Vacuolation, seminiferous tubule		-	-	1	-	-
Epididymis							
	Cell infiltration, interstitial		-	-	-	-	-
	Debris, epididymal tubule		-	-	P	-	-
Sciatic nerve			-	-	-	-	-

-:Not remarkable or none 1:Minimal 2:Mild 3:Moderate 4:Severe P:present

Table S1 Related to Figure 3. Individual histopathological findings in rats

Observation Parameter	Implantation substance / Animal No.	Hydrogel glucose sensor entry 1	entry 2	entry 3	entry 4	entry 5	Polyurethane entry 6	entry 7	entry 8	entry 9	entry 10
Cell type / response [b]											
1) Polymorphonuclear cells		1	1	1	1	1	1	1	1	1	2
2) Lymphocytes		2	2	2	2	2	2	2	2	2	3
3) Plasma cells		1	1	1	1	1	1	1	1	1	1
4) Macrophages		2	2	2	2	2	1	1	1	1	1
5) Giant cells		0	0	0	0	0	1	1	1	1	1
6) Necrosis		0	0	0	0	0	0	1	1	1	1
Sub-total		6	6	6	6	6	6	7	7	7	9
	Average			6					7.2		
Other findings (response) [b]											
7) Neovascularization		1	1	2	2	2	1	1	2	1	2
8) Fibrosis		2	2	2	2	2	2	1	2	2	2
9) Fatty infiltrate		0	0	0	1	0	0	0	0	0	0
Sub-total		3	3	4	5	4	3	2	4	3	4
	Average			3.8					3.2		
Total		9	9	10	11	10	9	9	11	10	13
	Average			9.8					10.4		

a): Subcutaneous tissue in the section

b): See the tables of scoring criteria (ISO 10993-6:2007 (E))

Cell type/response

Cell type/response	Score 0	1	2	3	4
Polymorphonuclear cells	0	Rare, 1-5/phf*	5-10/phf	Heavy infiltrate	Packed
Lymphocytes	0	Rare, 1-5/phf	5-10/phf	Heavy infiltrate	Packed
Plasma cells	0	Rare, 1-5/phf	5-10/phf	Heavy infiltrate	Packed
Macrophages	0	Rare, 1-5/phf	5-10/phf	Heavy infiltrate	Packed
Giant cells	0	Rare, 1-2/phf	3-5/phf	Heavy infiltrate	Sheets
Necrosis	0	Minimal	Mild	Moderate	Severe

Response

Response	Score 0	1	2	3	4
Neovascularization	0	Minimal capillary proliferation, focal, 1-3 buds	Groups of 4-7 capillaries with supporting fibroblastic structures	Broad band of capillaries with supporting structures	Extensive band of capillaries with supporting fibroblastic structures
Fibrosis	0	Narrow band	Moderately thick band	Thick band	Extensive band
Fatty infiltrate	0	Minimal amount of fat associated with fibrosis	Several layers of fat and fibrosis	Elongated and broad accumulation of fat cells about the implant site	Extensive fat completely surrounding the implant

*: phf = per high powered (400x) field

c): Thickness of inflammatory layer ; (①+②) / 2 x 0.025 mm (Scale of eyepiece micrometer)

Table S2 Related to Figure 3. Individual histopathological findings at subcutaneous implant sites and normal regions

Transparent Methods.

Fabrication of the plate-shaped hydrogels

The plate-shaped hydrogels were fabricated using the silicone mold and polyethylene terephthalate (PET) cover. The pregel solution for the hydrogel glucose sensors alone contained 10% w/v glucose-responsive fluorescent monomer(NARD research center.,) 15% w/v acrylamide (AAm) (FUJIFILM Wako Pure Chemical Industries Ltd.), 10% w/v Acryl-PEG (Sigma-Aldrich), 0.3% w/v *N, N'*-methylene-bis-acrylamide (Bis-AAm) (FUJIFILM Wako Pure Chemical Industries Ltd.), and 0.9% w/v sodium persulfate (SPS) (Kanto Chemical Co. Inc.) in a 60 mM phosphate buffer with 1.0 mM ethylenediaminetetraacetic acid (EDTA) (Nacalai Tesque Inc.), pH 7.4. In addition, the pregel solution for the hydrogel glucose sensors with antioxidant enzymes contained 0.1% w/v catalase (FUJIFILM Wako Pure Chemical Industries Ltd.), and 0.1% w/v superoxide dismutase (FUJIFILM Wako Pure Chemical Industries Ltd.). The pregel solution containing *N, N, N', N'* -tetramethylethylenediamine (TEMED) (FUJIFILM Wako Pure Chemical Industries Ltd.) was poured into the silicone mold and then stored at 37 °C. After 30 min, the hydrogel glucose sensors were removed from the silicon mold. They were then washed with Milli-Q® water for > 24 hours to remove unreacted monomers.

Characterization

The fluorescence study was performed on a microplate reader (Cytation 5, BioTek). We inserted the hydrogel plates in a 1-mL microtube with glucose saline solution at 0, 100, 300, 500, 1000 mg dL^{-1} at room temperature. After 30 min, the PDMS well of microplate were filled with the hydrogel plate and 100 μL of saline was added with each glucose concentration. We measured of the fluorescence intensity using an excitation wavelength of 365 nm and an emission wavelength of 492 nm. FT-IR spectra were recorded on a Fourier-transform infrared spectrometer (FT/IR-6100/ATR PRO450-S, JASCO) with xerogel. The water in the hydrogel plate was evaporated by a vacuum pump before measuring.

Fabrication of the implantable fluorescent device

The implantable fluorescent devices for CGM were composed of the implantable unit, a wire module, and the rechargeable battery. The implantable unit was constructed based on a structure with the Light Photo Sensor (APDS-9005, Avago) as a photodetector and UV-LED (SM1206 UV-405-IL, Bivar) as the excited light source. We adjusted the light output power to between 500 and 600 μW using the light intensity meter.

Histopathological examination

The histopathological examinations were carried out at a contract research organization, the Bozo Research Center Inc., Tokyo, Japan, in accordance with the guidelines of the Duration of chronic toxicity testing in animals (ICH S4). Each specimen of skin, sciatic nerve, testis, epididymis, and liver is embedded in paraffin, then sliced, stained with hematoxylin and Eosin, and observed under an optical microscope.

Continuous glucose monitoring

All animal experiments in this study were approved in advance by the Animal Care Committee at the University of Tokyo (approval number: 26-4). All animal care and experiments complied with the Guide for Animal Use and Care published by the University of Tokyo. Six-week-old male Sprague Dawley rats were purchased from Japan SLC (Shizuoka, Japan). They had free access to food (CE-2: CLEA Japan, Inc., Tokyo, Japan) and water in an animal room that was maintained at 23 ± 2 °C with a 12-h light–dark cycle. Following this 1-week conditioning period, the rats underwent surgical procedures as follows: the rat was anesthetized and had two polyurethane catheters inserted; one catheter was inserted into its right jugular vein and the other into its left carotid artery. After that, the cannulated rats were housed for another 7 days for a post-surgical recovery period until they were used for experiments. After the recovery period, the

device was implanted into the subcutaneous space of rats and glucose concentrations were monitored continuously under anesthesia in rats. Blood glucose concentrations were measured using Accu-Chek® (Roche Diagnostics, Basel, Switzerland) from the caudate vein of rats.
